# P-1536. The Use of Ampicillin/sulbactam for Carbapenem-resistant *Acinetobacter baumannii* (CRAB) Infections: Does Combination Therapy Improve Clinical Outcomes?

**DOI:** 10.1093/ofid/ofae631.1704

**Published:** 2025-01-29

**Authors:** Kirby An, Thien-Ly Doan, Adam Grunseich, Barbara Kamel, Aya Haghamad, Jamie Lemon, Stefan Juretschko, Henry Donaghy

**Affiliations:** Northwell - Long Island Jewish Medical Center/North Shore University Hospital, New Hyde Park, NY; Long Island Jewish Medical Center, New Hyde Park, New York; Northwell - Long Island Jewish Medical Center, New Hyde Park, New York; Northwell Health, New Hyde Park, New York; Northwell Laboratories, Lake Success, New York; Northwell Health Laboratories, Lake Success, New York; Northwell Health laboratories, Little Neck, New York; Northwell Health, New Hyde Park, New York

## Abstract

**Background:**

The 2023 IDSA guidance on the Treatment of Antimicrobial Resistant Gram-negative Infections recommends combination therapy with ampicillin/sulbactam (Amp/S) for carbapenem-resistant *Acinetobacter baumannii* (CRAB) infections. The study purpose is to describe the prescribing patterns of Amp/S combination therapy versus monotherapy therapy for the treatment of CRAB infections. The primary endpoint of treatment failure is a composite of a change in therapy due to clinical worsening, persistent infection at 14-days, or attributable mortality from invasive infection. Secondary objectives are to evaluate outcome measures.

Baseline Characteristics

**Figure:**
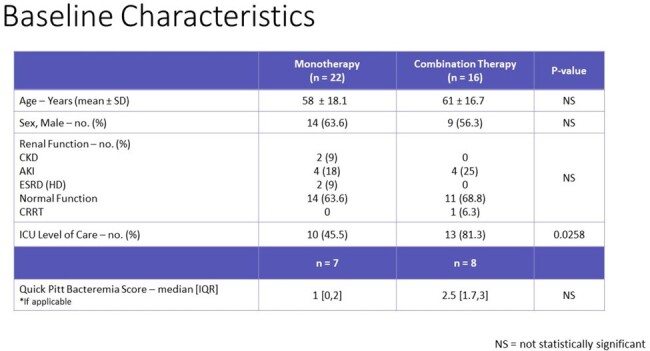

Patients well-matched with the exception of ICU level of care, where the combination therapy group was increased.

**Methods:**

This IRB-approved, retrospective observational chart review evaluated those admitted at a Northwell Health with a culture (blood, urine, wound, lower respiratory tract) positive with CRAB. Adult patients were included if they received at least 72 hours of Amp/S monotherapy or in combination with other agents from 1/2017 to 12/2023. Patients were excluded if they expired/discharged within 72 hours of treatment. Data collected included demographics, treatment received (agents, doses, duration) and hospital length of stay (LOS). Descriptive statistics were utilized.

Outcomes - Primary and Secondary Outcomes

**Figure:**
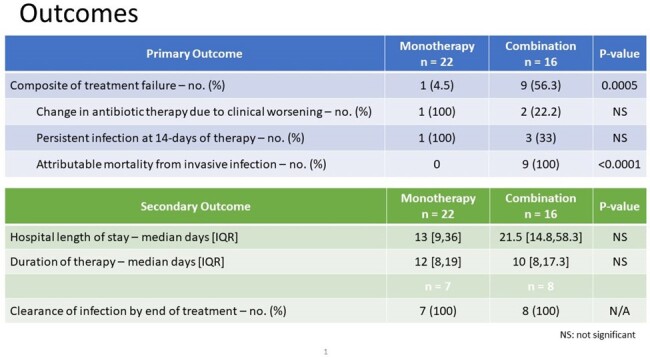

Treatment failure was seen more in the combination therapy group, which was driven by attributable mortality from invasive disease. There was no difference between the groups in terms of hospital length of stay or duration of antimicrobial therapy.

**Results:**

A total of 295 patients were screened, of which 38 were included in the study. Blood cultures were most prevalent in 39.5%, followed by wound (31.6%). Most received monotherapy (57.9%) while 42.1% had combination therapy. Combination therapy was used more often when the isolate was non-susceptible (83.3% vs.to 5% when sensitive). More patients received standard compared to high-dose Amp/S (31.7% vs. 68.4% respectively). Duration of therapy was similar. One patient on monotherapy was escalated to combination of therapy. Treatment failure was seen in 9 patients in the combination therapy. The median LOS was greater in the combination therapy than monotherapy (21.5 vs. 13 days).

Results - Primary and Secondary Outcomes

**Figure:**
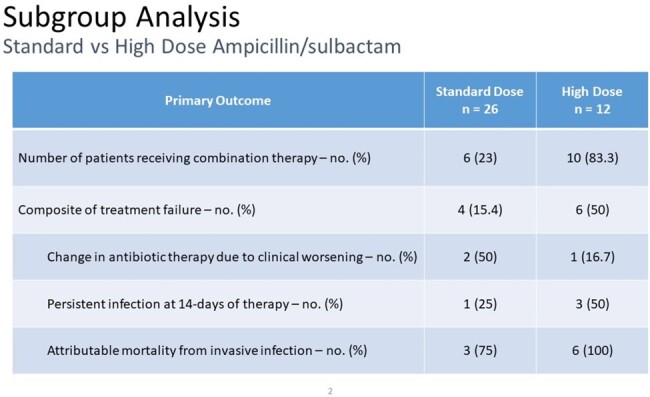

More patients receiving high dose ampicillin/sulbactam were treated with combination therapy. More treatment failure was seen in the high-dose ampicilin/sulbactam group, but that may be due to selection bias (e.g., more critically ill patients received more aggressive therapy using combinations of antibiotics).

**Conclusion:**

The prescribing patterns at Northwell Health for CRAB infections are varied and do not adhere to the IDSA guidance for CRAB infections. Most of the patients in the combination therapy had longer LOS, which may be due to selection bias in terms of disease severity. Education regarding the IDSA guidance may help steer prescribers to utilize more combination therapy with higher doses of Amp/S,

Subgroup Analysis of Susceptible vs. Non-susceptible Isolates

**Figure:**
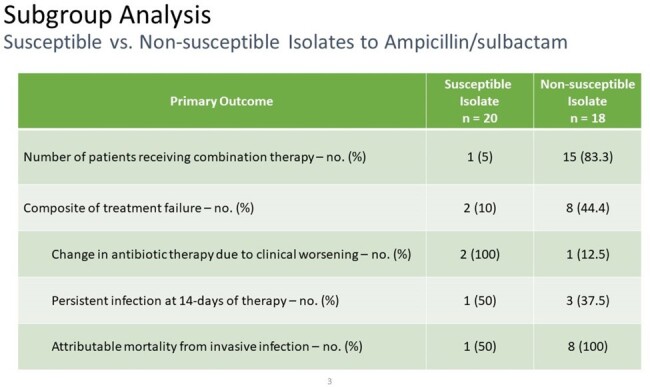

More patients with non-susceptible isolates received combination therapy.

**Disclosures:**

**All Authors**: No reported disclosures

